# Postnatal epigenetic reprogramming in the germline of a marsupial, the tammar wallaby

**DOI:** 10.1186/1756-8935-6-14

**Published:** 2013-06-03

**Authors:** Shunsuke Suzuki, Geoffrey Shaw, Marilyn B Renfree

**Affiliations:** 1 Department of Zoology, The University of Melbourne, Victoria, 3010, Australia; 2 Epigenomics Division, Frontier Agriscience and Technology Center, Faculty of Agriculture, Shinshu University, Nagano, 399-4598, Japan

**Keywords:** Epigenetic reprogramming, Genomic imprinting, Marsupial germ cells, Germ cell methylation

## Abstract

**Background:**

Epigenetic reprogramming is essential to restore totipotency and to reset genomic imprints during mammalian germ cell development and gamete formation. The dynamic DNA methylation change at DMRs (differentially methylated regions) within imprinted domains and of retrotransposons is characteristic of this process. Both marsupials and eutherian mammals have genomic imprinting but these two subgroups have been evolving separately for up to 160 million years. Marsupials have a unique reproductive strategy and deliver tiny, altricial young that complete their development within their mother's pouch. Germ cell proliferation in the genital ridge continues after birth in the tammar wallaby (*Macropus eugenii*), and it is only after 25 days postpartum that female germ cells begin to enter meiosis and male germ cells begin to enter mitotic arrest. At least two marsupial imprinted loci (*PEG10* and *H19*) also have DMRs. To investigate the evolution of epigenetic reprogramming in the marsupial germline, here we collected germ cells from male pouch young of the tammar wallaby and analysed the methylation status of *PEG10* and *H19* DMR, an LTR (long terminal repeat) and a non-LTR retrotransposons.

**Results:**

Demethylation of the *H19* DMR was almost completed by 14 days postpartum and *de-novo* methylation started from 34 days postpartum. These stages correspond to 14 days after the completion of primordial germ cell migration into genital ridge (demethylation) and 9 days after the first detection of mitotic arrest (re-methylation) in the male germ cells. Interestingly, the *PEG10* DMR was already unmethylated at 7 days postpartum, suggesting that the timing of epigenetic reprogramming is not the same at all genomic loci. Retrotransposon methylation was not completely removed after the demethylation event in the germ cells, similar to the situation in the mouse.

**Conclusions:**

Thus, despite the postnatal occurrence of epigenetic reprogramming and the persistence of genome-wide undermethylation for 20 days in the postnatal tammar, the relative timing and mechanism of germ cell reprogramming are conserved between marsupials and eutherians. We suggest that the basic mechanism of epigenetic reprogramming had already been established before the marsupial-eutherian split and has been faithfully maintained for at least 160 million years and may reflect the timing of the onset of mitotic arrest in the male germline.

## Background

Genome-wide dynamic changes of epigenetic states during mammalian germ cell development, called epigenetic reprogramming, are essential to restore totipotency and to renew parental imprinting in the male and female germ cells [1-4]. In mice, loss of DNA methylation and histone H3 lysine 9 dimethylation (H3K9me2) followed by the gain of H3K27me3 are the first gross epigenetic changes observed in migrating primordial germ cells (PGCs) between E7.5 and E9.5 [5,6]. Then, the second wave of DNA demethylation which is associated with the erasure of parental imprinting, promoter methylation of germline genes and with the reduction of retrotransposon methylation takes place around E11.5, just after PGCs have entered into the genital ridges [7-10]. From E14.5, *de-novo* DNA methylation dependent on the actions of the DNMT3 family re-establishes paternal imprints and methylation of retrotransposons in G1-arrested male germ cells, known as prospermatogonia or male gonocytes [11-18].

In higher vertebrates, genomic imprinting has been identified in eutherian and marsupial mammals [19-24]. However, of the 16 or so eutherian imprinted genes examined so far in marsupials, only six are imprinted [23-35]. Furthermore, there are only two DMRs, associated with *PEG10* and *H19*, that have been discovered so far, in marsupials, both in the tammar wallaby [24,30]. The tammar *H19* DMR was identified as a germline DMR because it was fully methylated in adult testes [30]. However, the precise timing of epigenetic reprogramming in the developing germ cells of marsupials has never been established. Eutherians and marsupials have been evolving separately for up to 160 million years [36]. Marsupials have a unique reproductive strategy and deliver tiny, altricial young that complete their development within their mother’s pouch [37]. In the tammar, most PGCs complete their migration to the genital ridges just before birth [38]. Post-migratory PGCs continue to proliferate after birth, and it is only after 25 days postpartum that female germ cells begin to enter meiosis while male germ cells enter into G1-phase mitotic arrest [39,40]. To compare the evolution of epigenetic reprogramming between this distantly related mammal and the mouse, we analysed the methylation dynamics of the *H19* DMR, which is the only paternal DMR discovered in marsupials so far, an LTR and a non-LTR retrotransposons in the male germline of the tammar wallaby during the postnatal proliferation and early mitotic arrest stages.

## Results and discussion

### Isolation of germ cells from the tammar pouch young testes

**Figure 1:**
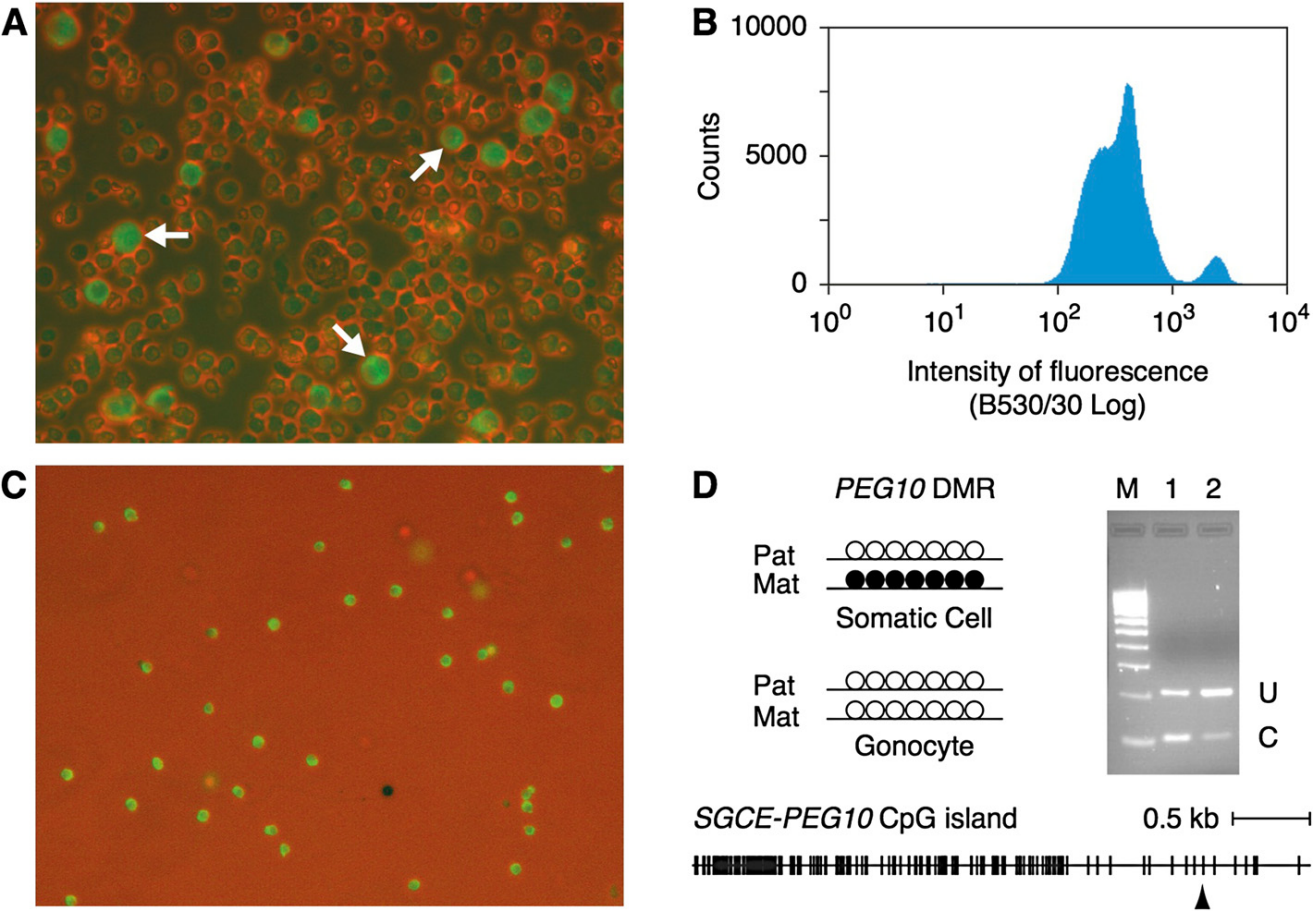
**Evaluation of the purity of the presumed germ cells isolated by FACS.** (**A**) Single cell suspension of the D28 tammar pouch young testes labeled by the DDX4/VASA antibody. The white arrows indicate some of presumed germ cells showing the stronger fluorescence. (**B**) A result of FACS showing that the presumed germ cells with the stronger fluorescence are separable from the rest of cell populations in which some low level fluorescence was detectable. (**C**) The content of the presumed germ cell fraction after FACS showing the strong fluorescence in the most abundant cells. (**D**) Left: Illustration of the virtual DNA methylation pattern of the *PEG10* DMR (a known maternally methylated DMR) in somatic cells and gonocytes (which should not be methylated in male germline). The white and black circles represent unmethylated and methylated CpG sites, respectively. Right: Result of COBRA (combined bisulphite and restriction analysis) at the *PEG10* DMR. U and C bands are the uncut and cut bands indicating the amount of unmethylated and methylated alleles, respectively. Lane M, size marker. Lane 1, COBRA using the genomic DNA extracted from kidney (control somatic tissue). Lane 2, COBRA using the genomic DNA extracted from the presumed germ cells collected by FACS. Bottom: The vertical bar represent a single CpG site in the *SGCE-PEG10* CpG island and the location of CpG site used for COBRA is indicated by the arrow head.

To obtain genomic DNA derived from germ cells, we separated germ cells from the single cell suspension of pouch young testes by FACS (fluorescence activated cell sorting). For the labeling of germ cells, we used the antibodies against mouse DDX4 (DEAD (Asp-Glu-Ala-Asp) box polypeptide 4)/VASA and SSEA1 (stage specific embryonic antigen 1) that we have previously evaluated the specific reactivity for tammar DDX4/VASA and SSEA1 orthologues and the specific staining of tammar germ cells [41,42]. The DDX4/VASA antibody was used for cells isolated from animals older than 14 days postpartum and the SSEA1 antibody was used for experiments before 14 days postpartum. Using the DDX4/VASA antibody, we confirmed that a distinct subpopulation of cells clearly showed brighter fluorescence than the rest of population in which the subtle fluorescence is still detectable (Figure 1A). The cells with brighter fluorescence were successfully separated by FACS and we confirmed that most of the collected cells were strongly fluorescent (Figure 1B, C). To confirm if the collected cells were predominantly germ cells, we checked the DNA methylation level of the *PEG10* DMR by COBRA (combined bisulphite restriction analysis) using genomic DNA extracted from the collected cells. Because the *PEG10* DMR is a maternally methylated DMR, it should be unmethylated in male germ cells (Figure 1D left). The result of COBRA using the collected cells showed the only faint cut band which appears when the AciI recognition site is methylated while the control somatic tissue (kidney) showed similar intensities of the cut and uncut bands as expected, suggesting that the somatic cell contamination in the collected cells was limited even if all the cut band was derived from somatic cells (Figure 1D right). Hence we used genomic DNA prepared by this way to the following analyses.

### DNA demethylation in the germline of tammar male pouch young

**Figure 2:**
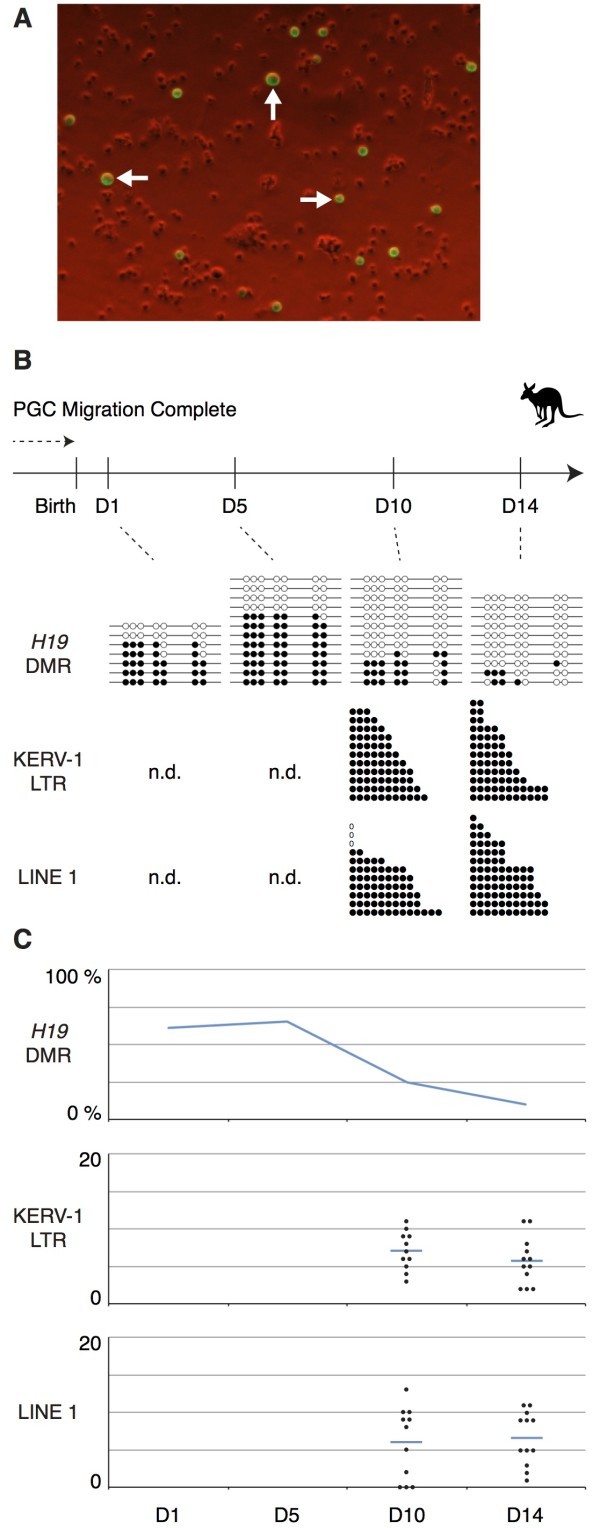
**DNA methylation status of the *H19 *DMR, KERV-1 LTR and LINE1 5**′ **region in male pouch young germ cells at 1, 5, 10 and 14 days postpartum.** (**A**) Single cell suspension of the D5 tammar pouch young gonads labeled by the SSEA1 antibody. The white arrows indicate some of presumed germ cells labeled very clearly. (**B**) The black and white circles represent methylated and unmethylated CpG sites, respectively. The KERV-1 and LINE1 data only show the number of methylated CpG in each sequence as CpG sites are not always conserved among clones because of the heterogeneous amplification of these repetitive sequences. No data for ‘n.d.’. (**C**) The vertical axis for the graph of *H19* DMR methylation represents the percentage of methylated CpG sites based on the data shown in Figure 2B. In the graphs of retrotransposon methylation, the vertical axes represent total number of methylated CpG sites in each sequence instead of percentage. Blue horizontal bars indicate average of the data.

**Figure 3:**
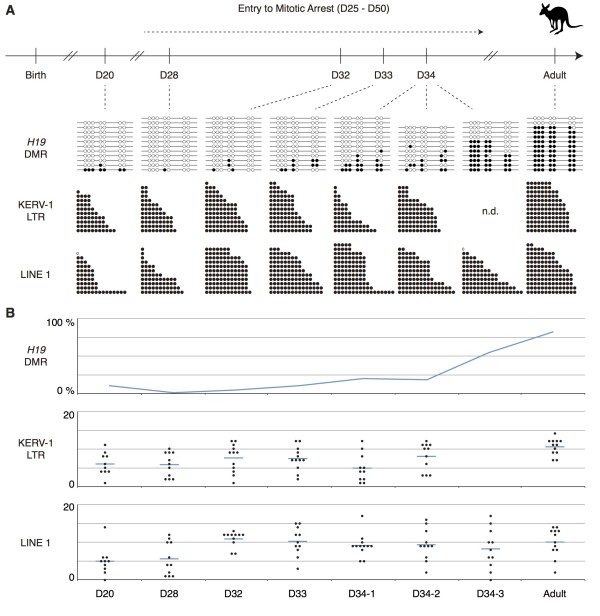
**DNA methylation status of the *H19 *DMR, KERV-1 LTR and LINE1 5**′ **region in male pouch young germ cells at 20, 28, 32, 33 and 34 days postpartum and in adult testis.** (**A**) The black and white circles represent methylated and unmethylated CpG sites, respectively. The KERV-1 and LINE1 data only show the number of methylated CpG in each sequence as CpG sites are not always conserved among clones because of the heterogeneous amplification of these repetitive sequences. No data for ‘n.d.’. (**B**) The vertical axis for the graph of *H19* DMR methylation represents the percentage of methylated CpG sites based on the data shown in Figure 3A. In the graphs of retrotransposon methylation, the vertical axes represent total number of methylated CpG sites in each sequence instead of percentage. Blue horizontal bars indicate average of the data.

**Figure 4:**
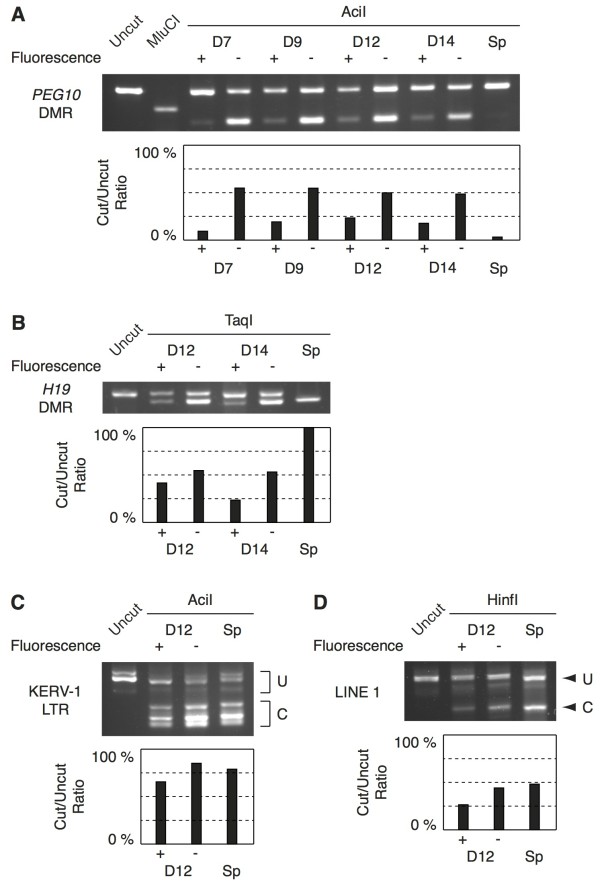
**DNA methylation analysis by COBRA for the *PEG10 *and *H19 *DMRs, KERV-1 LTR and LINE1 5**′ **region in male pouch young germ and somatic cells at 7, 9, 12 and 14 days postpartum and in adult sperm.** The gel pictures show the cut and uncut bands after digestion by the restriction enzymes indicated in each panel. The fluorescence positive and negative samples represent positively sorted presumed germ cells and negatively sorted somatic control cells. Adult sperm samples were labeled as ‘Sp’. The vertical axes of bar graphs represent ratio of the intensity of the cut bands reflecting the methylation level of each sample. In C and D, the regions and bands subjected to cut/uncut intensity calculation were labeled by U for uncut and C for cut, respectively. (**A**) *PEG10* DMR, (**B**) *H19* DMR, (**C**) KERV-1 LTR, (**D**) LINE1 5′ region.

To investigate how DNA methylation levels change during male germ cell development in the tammar, we first determined when the demethylation occurs in tammar PGC development by analysis of the *PEG10* and *H19* DMR, an LTR of KERV-1 and the 5′ region of LINE1 as an example of LTR and non-LTR retrotransposons, respectively. The monoclonal mouse SSEA1 antibody was used to label early germ cells before the start of DDX4/VASA expression (which does not begin until around day 10 postpartum [41]). The SSEA1 antibody gave a clear strong fluorescence of cells without any detectable non-specific reactivity to other cell populations (Figure 2A), consistent with previous observations of specific staining to tammar PGCs using this SSEA1 antibody [42]. The DNA samples extracted from these cells were used for the methylation analysis. While the *H19* DMR was still differentially methylated at 1 day and 5 days postpartum, it had an intermediate level of methylation at 10 days postpartum and was nearly fully demethylated by 14 days postpartum (Figure 2B, C). The bisulphite conversion method used in this study would not provide the information about 5-hydroxymethylcytosine [43-45]. Recently, Hackett *et al.* reported that erasure of CpG methylation in mouse PGCs occurs via conversion to 5-hydroxymethylcytosine [46]. Therefore, it will be important to confirm whether the dynamics of hydroxymethylation are also conserved between marsupials and eutherians in future studies. The methylation level of both LTR and non-LTR retrotransposons at 14 days postpartum was clearly lower than in the adult testis, but they were not completely unmethylated, unlike the *H19* DMR (Figures 2B and 3A). This is similar to the observations in the mouse that some degree of methylation was retained in the IAP, Line1 and SineB1 loci in murine germ cells in the undermethylated state [7,8,15]. To confirm these results using a more quantitative method, we performed a COBRA assay for these three genomic loci and the maternally methylated *PEG10* DMR using independently prepared samples and using primers amplifying different region in the *H19* DMR (Figure 4). Interestingly, the *PEG10* DMR was already unmethylated at 7 days postpartum, suggesting the timing of epigenetic reprogramming is not the same at all genomic loci (Figure 4A). The undermethylated state of *PEG10* DMR at these stages also confirmed that the purity of germ cells was reasonably consistent and at a similar level as the older age shown in Figure 1D, even when the smaller volumes of tissues from the younger animals were used. The complete conversion after bisulphite treatment was shown by the complete MluCI digestion (digests AATT sites that are created only after the bisulphite conversion in the amplified *PEG10* DMR fragment) and by the almost undetectable cut band in the adult sperm sample (Figure 4A). The *H19* DMR was still methylated at 12 days postpartum, but methylation was clearly reduced by 14 days postpartum (Figure 4B). These results are consistent with the data of bisulphite sequencing shown in Figure 2, excluding the possibility that there was a huge cloning bias in the bisulphite sequence data. The methylation levels of the retrotransposons were clearly lower in 12 days postpartum germ cells comparing the somatic cells and adult sperm (Figure 4C, D). Although we were not able to determine the precise timing of the demethylation of the *PEG10* DMR, both the sequencing and COBRA data suggest that the demethylation event at the *H19* DMR starts around 10 days postpartum and is almost completed by 14 days postpartum.

### *De-novo* DNA methylation in the germline of tammar male pouch young

We next determined when *de-novo* methylation took place at the *H19* DMR and the retrotransposons. At 20, 28, 32 and 33 days postpartum, the *H19* DMR was still nearly fully unmethylated, suggesting that the undermethylated states observed at 14 days postpartum had persisted at least until these stages (Figure 3A). At the same time, these data demonstrate that the effect of somatic cell contamination during germ cell separation to the results of methylation analyses was negligible, so we assume the faint cut bands in the *PEG10* DMR COBRA in Figures 1 and 4 may not be a simple reflection of somatic cell contamination. Also the methylation level of both LTR and non-LTR retrotransposons at 20 and 28 days postpartum was similar to that at 14 days postpartum (Figures 2B and C and 3A and B). On the other hand, we detected *de-novo* DNA methylation of the *H19* DMR in three different animals at 34 days postpartum, indicating that 34 days postpartum is the critical stage for the acquisition of *de-novo* methylation and that it occurs rapidly. The increase of methylation in the retrotransposons was detected at 32 days postpartum, two days earlier than *de-novo* methylation of the *H19* DMR (Figure 3A, B). It is possible that the methylation machinery responds more quickly to the retrotransposons retaining some degree of methylation than the fully unmethylated *H19* DMR. Alternatively, the methylation machinery might be slightly differently recruited to the *H19* DMR and to the retrotransposons. The G1-phase entry into mitotic arrest begins only after 25 days postpartum in the tammar male germline and is not complete until after day 50. Considering that germ cell development in the tammar wallaby takes much longer than in mouse and occurs postpartum, the relative timing and pattern of *de-novo* DNA methylation in the male germ cell development as well as the timing of demethylation is remarkably similar in both species. In mouse male germ cells undergoing mitotic arrest, NANOS2 maintains their arrested state and induces male-type germ cell differentiation including the expression of DNMT3L, an essential factor for the establishment of paternal imprinting and retrotransposon methylation [47]. The orthologue of *NANOS2* is found in the tammar genome (Hickford and Renfree, unpublished). Although the precise molecular pathway between NANOS2 and DNMT3L expression is still largely unknown, the similar relative timing of *de-novo* DNA methylation in the male germline of tammar and mouse, which starts shortly after the entry into mitotic arrest in both species, suggests that the molecular basis connecting these events has been conserved between marsupials and eutherians. The orthologues of the factors essential for paternal imprinting establishment in the mouse germline, such as DNMT3A, DNMT3L and BORIS/CTCFL, are also present in marsupials [48,49]. These orthologues most likely play the same critical role to establish the methylation imprint in the marsupial *H19* DMR, which occurs at a similar relative time in the male germ cell development as in that of the mouse.

**Figure 5:**
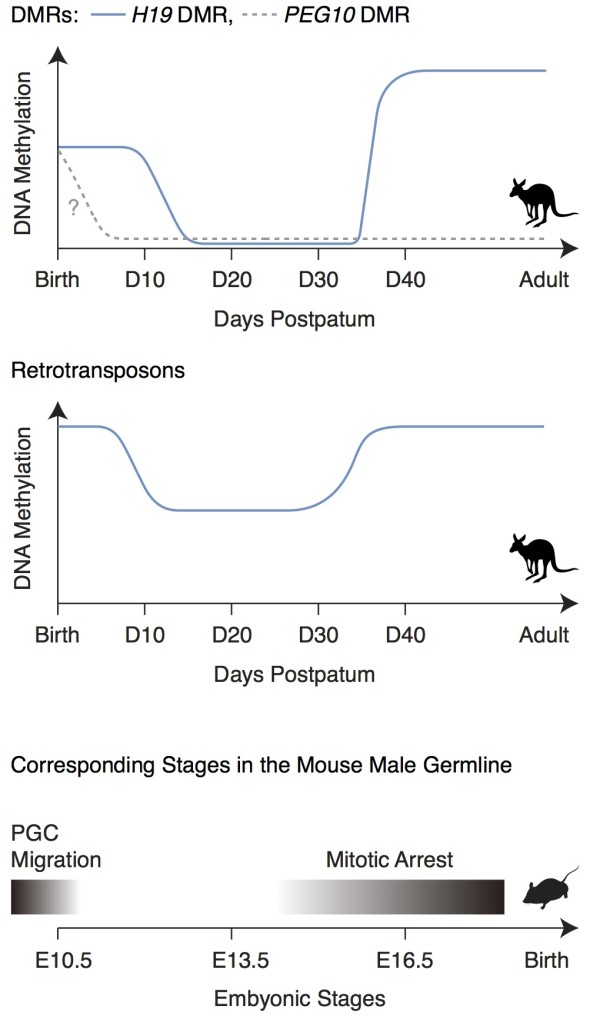
**Predicted DNA methylation dynamics during epigenetic reprogramming in the male germline of the tammar wallaby and corresponding stages in the mouse male germline.** The vertical axes represent relative DNA methylation level and the horizontal axes represent days postpartum. In the top graph, the predicted methylation dynamics of the *H19* and *PEG10* DMRs were represented by the blue and broken grey lines, respectively. Demethylation of the *H19* DMR was completed by 14 days postpartum and *de-novo* methylation started from 34 days postpartum. Retrotransposon methylation was not completely removed after the demethylation event in the germ cells, similar to the situation in the mouse. The corresponding stages in the mouse male germline were illustrated in the bottom. Thus, despite the occurrence of epigenetic reprogramming postnatally and the persistence of genome-wide undermethylation for 20 days in the postnatal tammar, the relative timing of germ cell reprogramming was conserved between marsupials and eutherians.

According to the timing of demethylation and *de-novo* methylation in the tammar germline, which occurred around 14 and 34 days postpartum, respectively, it is clear that tammar germ cells are exposed to the undermethylated state for about 20 days (Figure 5). In the mouse germline, some aspects of the piRNA (Piwi-interacting RNA) pathway related to post-transcriptional silencing such as mRNA cleavage, is one candidate to play a crucial role in retrotransposon inactivation from the onset of the undermethylated state until re-methylation occurs [50,51]. *Miwi, Miwi2, Mili* and *Ddx4/Vasa* are essential components in the mouse piRNA pathway [52-56]. Because piRNAs exist in the tammar testes [57] and all the orthologues of these four genes are found in the tammar genome (Hickford *et al*., 2011; S. Suzuki, unpublished), marsupials also possibly use the piRNA pathway to inactivate retrotransposons during the period when germ cells are undermethylated. For another candidate mechanism involves *Tex19.1* which regulates activity of a class of endogenous retroviruses by a post-transcriptional mechanism distinct from the piRNA pathway in the mouse male germline [51,58]. Unless the partial DNA methylation remaining while they are in the undermethylated state is enough to repress retrotransposons, any of these DNA methylation-independent mechanisms must be stable enough to inactivate retrotransposons for at least 20 days. It is likely that the marsupial orthologues of these factors are expressed in germ cells during this time but this awaits future confirmation.

## Conclusions

Demethylation and *de-novo* methylation in the male germline of a marsupial occurs over a prolonged period postpartum. Despite the occurrence of epigenetic reprogramming postnatally and the persistence of genome-wide undermethylation for 20 days in the postnatal tammar, the relative timing and mechanism of germ cell reprogramming was conserved between marsupials and eutherians. We suggest that the basic mechanism of epigenetic reprogramming had already been established before the marsupial-eutherian split and has been faithfully maintained for at least 160 million years and that it is tightly correlated with the onset of mitotic arrest in the male tammar wallaby.

## Methods

### Animals and tissue collection

Tammar wallabies (*Macropus eugenii*) of Kangaroo Island origin were maintained in our breeding colony in grassy, outdoor enclosures. Lucerne cubes, grass and water were provided *ad libitum* and supplemented with fresh vegetables. Gonads or testes were collected from pouch young aged between 1 and 34 days postpartum. The pouch young age was determined by plotting head length against growth curves for the tammar [59]. Experimental procedures conformed to Australian National Health and Medical Research Council (2004) guidelines and were approved by the Animal Experimentation Ethics Committees of the University of Melbourne.

### Preparation of single cell suspension

Gonads or testes were torn using a needle in 0.25% Trypsin/EDTA (Invitrogen) and were incubated for 10 min at 37°C. The gonadal/testicular cells were dissociated by 30 pipetting strokes with 1 mL plastic tips followed by 10 strokes with 200 μL plastic tips. The cell samples were passed through 40 μm cell strainer (BD Biosciences).

### Germ cell labeling

The cells were fixed in 4% PFA/PBS for 20 min at room temperature and then permeabilised in 0.1% Triton X-100/PBS for 15 min at room temperature. The primary antibody reactions were performed in 0.1% BSA and 0.05% Tween 20/PBS containing the SSEA1 antibody (1/30 of total reaction volume, MC-480; Developmental Studies Hybridoma Bank at the University of Iowa) or the DDX4/VASA antibody (1/300 of total reaction volume, ab13840; Abcam) for 30 min at room temperature. The cells were washed in 0.1% Tween 20/PBS and were labeled by the secondary antibodies (Invitrogen) in the same solution as the primary antibody reaction. The labeled single cell suspension samples were passed through 40 μm cell strainer (BD Biosciences) before fluorescence activated cell sorting, FACS (MoFlo Cell Sorter, Beckman Coulter and FACS Aria III, BD Biosciences).

### DNA methylation analyses

Genomic DNA was extracted from the germ cells collected by FACS using a Wizard Genomic DNA Purification Kit (Promega). Purified genomic DNA was treated with a sodium bisulphite solution as described previously [60,61]. After the bisulphite treatment for the genomic DNA, 30 to 38 cycles of PCR with the genomic DNA templates corresponding to 100 to 5,000 cells were carried out using the following primer pairs.

*PEG10* DMR Forward: 5′- CCTCCCATTAACTTTAAAATCACC -3′

*PEG10* DMR Reverse: 5′- ATTGTAGTAATGGGGTAGGTTATG -3′

*H19* DMR Forward: 5′- GAATGGGTTAGATGAGGGTAGTATAG -3′

*H19* DMR Reverse: 5′- TATCAAACACCAAAACCACAAATAA -3′

*H19* COBRA Forward: 5′- TTATTTTGGAGAAAATTTGAAGATAAGTAG -3′

*H19* COBRA Reverse: 5′- TATCCTAAAACATCAAAACCTAAATTAAAC -3′

KERV-1 LTR Forward: 5′- TAAACTCAATTCCATATAAACAATCTC -3′

KERV-1 LTR Reverse: 5′- TTTTTGTTTTGTAAGGGTTTTTTAG -3′

LINE1 Forward: 5′- GGAGATTTTTGTTTTAGAGAGATTTGTAAA -3′

LINE1 Reverse: 5′- TATAAAAACACCCCACTCCCCTCTC -3′

The PCR products for COBRA (combined bisulphite and restriction analysis) were digested with 1 to 10 units of MluCI, AciI, TaqI (New England Biolabs) or HinfI (TaKaRa) restriction enzymes for 2-3 h at 37°C or 65°C for TaqI. The intensity of the cut and uncut bands was quantified by ATTO CS Analyzer 3 software (ATTO). The PCR products for *H19* DMR and retrotransposons were cloned, and the clones were sequenced. The sequence data were analysed by QUMA (quantification tool for methylation analysis; http://quma.cdb.riken.jp) [62].

## Competing interests

The authors declare that they have no competing interests.

## Authors’ contributions

SS carried out the molecular and cellular studies, participated in the experimental design and data analysis, and drafted the manuscript. GS and MBR conceived of the study, and participated in its design and coordination and helped to draft the manuscript. All authors read and approved the final manuscript.
